# Mesenchymal stem cells for restoring endometrial function: An infertility perspective

**DOI:** 10.1002/rmb2.12339

**Published:** 2020-07-20

**Authors:** Ruttachuk Rungsiwiwut, Pramuan Virutamasen, Kamthorn Pruksananonda

**Affiliations:** ^1^ Department of Anatomy Faculty of Medicine Srinakharinwirot University Bangkok Thailand; ^2^ Department of Obstetrics and Gynecology Faculty of Medicine Chulalongkorn University Bangkok Thailand

**Keywords:** assisted reproductive medicine, cell therapy, endometrium, infertility, stem cells

## Abstract

**Background:**

Mesenchymal stem cells (MSCs) can be derived from several tissues such as bone marrow, placenta, adipose tissue, or endometrial tissue. MSCs gain a lot of attention for cell‐based therapy due to their characteristics including differentiation ability and immunomodulatory effect. Preclinical and clinical studies demonstrated that MSCs can be applied to treat female infertility by improving of the functions of ovary and uterus. This mini‐ review focuses on the current study of treatment of endometrial infertility by using MSCs.

**Methods:**

The present study performed a literature review focusing on the effect of MSCs for treatment of women infertility caused by endometrial dysfunction.

**Results:**

Bone marrow‐, umbilical cord‐, adipose‐, amniotic‐, and menstruation‐derived MSCs enhance endometrial cell proliferation, injury repairs as well as reducing scar formation. The beneficial mechanism probably via immunomodulatory, cell differentiation, stimulates endometrial cell proliferation and down‐regulation of fibrosis genes. The major advantage of using MSCs is to improve endometrial functions resulting in increased implantation and pregnancy.

**Conclusions:**

MSCs exhibit a potential for endometrial infertility treatment. Adipose‐ and menstruation‐derived stem cells show advantages over other sources because the cells can be derived easily and do not causes graft rejection after autologous transplantation.

## Graphical abstract

Mesenchymal stem cells for infertility treatment. A new insight into endometrial factor infertility management.

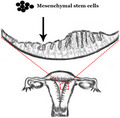

## 1 INTRODUCTION

### 1.1 Mesenchymal stem cells

Mesenchymal stem cells (MSCs) can be practically isolated from body tissues such as menstrual blood,^1^ adipose tissue,^2^ amniotic fluid,^3^ and endometrium.^4^ Based on their localization in different tissues, the researchers believe that MSCs might be involved in regulation of cells or tissue repair.^5^ The characteristics of MSCs derived from different source of tissues can be slightly different. For instance, MSCs derived from bone marrow, umbilical cord blood, adipose tissue, and placenta exhibit differences in immunophenotype, proliferation differentiation ability, and molecular signature.^6^ Interestingly, osteogenic differentiation of Mesenchymal stem cells derived from cord blood and placenta showed some variation according to the donor.^6^ Therefore, according to the minimal criteria proposed by the International Society for Cellular Therapy (ISCT) to define human MSCs are including, (a) under the standard culture condition, MSCs must grow adherently to surface of the culture vessels, (b) MSCs exhibit the phenotype similar to those fibroblast cells, (c) MSCs express at least CD73, CD90, and CD105 while lack expression of CD34, CD45, CD14, CD19, and HLA‐DR,^7^, ^8^ and (d) MSCs enable to osteogenic‐, chondrogenic‐, and adipogenic differentiation.^7^ Interestingly, MSCs have demonstrated the ability to differentiation into cardiomyocytes, ^9^ hepatocytes,^10^ and pancreatic secreting cells.^11^ Despite of the differentiation ability, the immunomodulatory capacity makes MSCs as a candidate cell type for immune therapy.^12^, ^13^, ^14^ The immunomodulatory mechanism of MSCs involves with migration of MSCs to the area of injury or inflammation, follows by interaction with immune cells resulting in suppression or stimulation of innate and adaptive immune system of the host.^15^ In addition, the ability of MSCs to downregulate of MHC class II upon allogeneic transplantation makes MSCs suitable for cell‐based therapy without the risk of immune rejection.^16^ With the advantage of MSCs, the first MSC drug was developed for immunomodulatory treatment of graft‐versus‐host disease (GVHD).^17^ Recently, the researchers in China have demonstrated the beneficial effect of MSCs after treatment of patients suffering from respiratory complication due to COVID‐19 infection.^18^, ^19^ COVID‐19 patients received intravenous infusions of MSCs and it improves the patient outcomes, indicates that the MSC treatment is safe and promising.

Therefore, several studies are focusing on using of MSCs as a high potential source of stem cells for regenerative medicine and immunomodulatory therapy in several diseases including infertility.

### 1.2 Endometrium

Human uterus is anatomically divided into three layers including perimetrium, myometrium, and endometrium. Endometrium locates at the innermost area of uterus which can be divided into two sublayers, the basal layer which connects to myometrium and the functional layer which is facing to the cavity of uterus.^20^ Endometrium is dynamically regenerated in the response to reproductive hormones. The regeneration of endometrium is influenced by the fluctuation of major hormonal levels including estrogen and progesterone, resulting in menstrual cycle.^21^ The menstrual cycle begins with the ovaries produces and releases estrogen.

Estrogen stimulates the endometrial cells to proliferate resulting in thicken of the functional layer. During this phase, the vessels and the endometrial glands are increasing their sizes. Later, in the mid of menstrual cycle, the peak of the estrogen level occurs followed by releasing of the oocytes so called ovulation. Following the ovulation, the level of progesterone begins to increase. Increasing of progesterone level activates the function of endometrial layer in order to prepare for implantation of the embryo. However, if the implantation does not occur, the levels of estrogen and progesterone will decline and followed by the shedding of endometrial lining. This shedding of endometrial tissue is recognized as menstruation. The cycle of menstruation is completed by finishing of shedding process and subsequently the new cycle begins. Therefore, regeneration of both functional and basal layers of endometrium importantly involves in menstruation and embryo implantation.

The process that the blastocyst embryo interacts with the functional layer of uterus is known as implantation. The process begins with the embryo attaches to the epithelial surface of endometrium, invades the epithelial surface, and proceeds decidualization. In general, successful clinical pregnancy depends on synchronization of blastocyst embryos and endometrial receptivity with optimal decidualization.^22^ Decidualization is the process in which the stromal cells of endometrium change their morphology in order to preparing the suitable conditions for embryo implantation.^22^ In human, however, decidua is formed routinely in relation to menstruation and shed off if the embryo implantation does not occur.^23^ In contrast, in case of fertilization followed by implanting of embryo, the decidua stroma remains cotact.^24^ It is generally accepted that decidualization plays a key role in embryo implantation and maintenance of pregnancy. During the process of decidualization, there are cellular and molecular changes occur in the endometrial layer include, increasing of oxidative stress resistance, modulating a local inflammatory response resulting in allowing of trophoblast invasion. In contrast, diminishing of decidualization causes some reproductive problems, such as failure of implantation, miscarriage, and dysfunction of uterus.^25^ Interestingly, low implantation rate after transferring of high‐quality embryos in infertile patients emphasizes the importance of a diminished decidualization. Therefore, impairment of decidualization is one of a major causes of pregnancy failure and infertility.^26^

### 1.3 Infertility

Infertility often creates problems for the couples, and the women are frequently blamed for the cause of problem.^27^ In a larger scale, however, infertility involves in decreasing of birthrate in many country and influences the national policy, especially in the country that is facing aging society. Infertility is clinically defined as a disease of the reproductive system, failure to achieve a clinical pregnancy after 12 months of having regular unprotected sexual intercourse.^28^ According to World Health Organization (WHO), infertility can be classified into primary and secondary infertility. Primary infertility is referred to a woman who has never been pregnant and secondary infertility is referred to a woman who has had at least one successful pregnancy but later experiences the incapability to pregnant.^29^ Mascarenhas and colleagues (2012)^30^ reported that globally, 19.2 million out of 48.5 million of infertile couple suffered from primary infertility and 29.3 million infertile couple were suffered from secondary infertility. Interestingly, primary infertility was a common cause for couple in the developed countries, whereas in developing countries, secondary infertility was found to be a major cause.^30^ Despite of the country factor, approximately 25% of infertility has been attributed to male, whereas 58% to female, and 17% to unexplained cause.^31^ Focusing on female factor, female infertility may cause by disturbances of reproductive organs or central nervous system that secrets hormones controlling the reproductive system. The abnormalities including anovulation, fallopian tube disease, pelvic adhesions, endometriosis, and unexplained factors are reported as the cause of female infertility.^32^ However, it is generally accepted that main factor affecting female fertility is an age. Theoretically, woman is born with limited number of oocytes. Female fertility dramatically declines after the certain age, due to the degeneration rate of follicles in the ovaries, increasing of abnormal mitochondria and chromosomal abnormalities in oocytes as well as increasing the incidence of miscarriage.^33^ Treatment of female infertility is still complicated; therefore, novel treatment is needed to be developed and evaluated for safety and efficiency.

### 1.4 Female infertility caused by endometrium diseases or abnormalities

As mentioned in the previous section, several factors cause female infertility including anovulation, fallopian tube disease, pelvic adhesions, endometriosis, and unexplained infertility.^32^ However, this review highlights only infertility that caused by endometrium dysfunction.

Fibroids or scaring in the uterine tissue can deform the uterine cavity resulting in difficulty for implantation. The major causes of scarring or adhesion include previous uterine surgery or infection of the endometrium. Intrauterine adhesions (IUAs), also known as Asherman's syndrome, caused fibrous strings connecting inside the uterine wall. Damage of the endometrial layer can lead to scar formation resulting in partially or completely obstructs the uterine cavity.^34^ Several abnormalities of the reproductive system including hypomenorrhea, amenorrhea, infertility, and recurrent miscarriages can be found in patients with IUAs.^34^ To date, numerous treatments such as hormonal treatment, insertion of an intrauterine device (IUD), or barrier gels can be applied for treatment of IUAs.^34^, ^35^ Interestingly, existing of stem cells in the basal layer of endometrium is probably the key factors for treatment of IUAs by stem cell‐based therapy.^36^

Endometriosis is a disease with unknown cause, characterized by ectopic endometrial implants throughout the pelvis, and negatively impacts fertility.^37^ Some patients that develop minimal to mild endometriosis have an almost normal probability of pregnancy but some experience infertility for unknown reasons. In cases of moderate and severe endometriosis, more scar adhesions occur and interrupt the movement of oocyte to the fallopian tube, causing the reduction of natural conception. As a result, endometriosis may lead to an abnormal implantation resulting in subfertility.^38^

Congenital mesonephric anomalies and Mullerian malformation results in uterine abnormalities. Anatomical abnormalities of female reproductive organs including duplication of uterus, cervix and vagina, failure of forming the uterus, and abnormalities of uterine structures cause serious clinical symptoms, affects the quality of life, and creates the fertility problems.^39^, ^40^ Endometrial thickness can be used to predict the endometrial receptivity.^41^ Endometrial thickness is clinically defined as the distance between the myometrium and endometrium which can be measured by ultrasonography.^42^ Normally, the endometrial thickness shows different ranges according to the uterine cycles. In menstrual phase, the thickness ranges from 1 to 4 mm, mid‐proliferative phase ranges from 4 to 8 mm, late follicular phase ranges from 8 to 14 mm, and secretory phase ranges from 7 to 14 mm.^43^, ^44^ Slow proliferation of uterine epithelium and stroma cells can lead to thin endometrium. Several pathological conditions such as Asherman's syndrome, previous intrauterine surgery, pelvic radiation have been reported to be the causes of thin endometrium.^45^, ^46^ Theoretically, thin endometrium can be improved by regeneration of endometrial stromal and epithelial cells resulting in improvement of implantation.^45^ Some reports suggested that the endometrium which represents the thickness more than 17 mm is related with improving pregnancy rate.^47^, ^48^ However, it has to be considered that thickened endometrium is associated with fibroid or polyps which have adversely affect the implantation of embryo.^49^ In order to solve the infertility caused by thin endometrium, several methods for endometrial preparation have been described. It is still in a premature stage to conclude for the best procedure for improving implantation of thin endometrial patient.

### 1.5 Current application of mesenchymal stem cells for infertility caused by endometrial dysfunction

Stem cells exhibit unique characteristics of self‐renewal and differentiation potential. Recently, cell therapy using stem cells becomes a novel treatment for several diseases including infertility. However, only the uses of MSCs for treatment of endometrial infertility are highlighted in the present review.

#### 1.5.1 Bone marrow‐derived stem cells

Bone marrow‐derived stem cells (BMSCs) can be differentiated into many cell types such as skeletal myoblasts, hepatic epithelium, neuroectodermal cells, and endometrial cells.^50^, ^51^ BMSCs display immunoregulatory properties.^52^ In addition, it has been demonstrated that BMSCs can secrete several growth factors such as hepatocyte growth factor, platelet‐derived growth factor, and transforming growth factor‐β.^53^ Recently, BMSCs have demonstrated the successful treatment of endometrial dysfunction in animal studies and clinical trials.

A study of Tal and colleagues (2019)^54^ provide the interesting evidence of mobilization of mouse MSCs from bone marrow to blood circulation during pregnancy. BMSCs were circulated to the decidual stroma and further differentiated into nonhematopoietic prolactin‐expressing decidual cells. Importantly, their results showed that nonhematopoietic BMSCs impact the decidual molecular niche and subsequently improve the implantation. Several studies confirm that BMSCs can migrate to the endometrium following systemic infusion. In the model of bone marrow transplantation between male to female mice, the results demonstrated that BMSC derived from donor male bone marrow migrates to the uterus of the female recipient mice. Cells expressing SRY gene and Y chromosome were detected by fluorescence in situ hybridization and immunofluorescence in the uterus of female mice confirms the migration ability of BMSCs.^51^ Similar to the results of mouse model, donor‐derived cells were identified in the uterine tissue of the woman who had undergone allogeneic bone marrow transplantation, confirms BMSC migratory effect.^55^, ^56^ Therefore, BMSCs may be a candidate cells for the reproductive cell replacement.

For animal applications, the previous studies show that after injection of BMSCs, various growth factors were secreted into the endometrium. The secreted growth factors effectively stimulate microvascular endothelial cell proliferation and differentiation.^57^, ^58^ Moreover, transplantation of BMSCs improves infertility of a mouse thin endometrium model by upregulating of markers for endometrial receptivity.^59^ The study of Zhao et al^60^ demonstrated that injection on BMSCs into uterine cavity elevates the expression of protein marker of endometrial cells resulting in increasing the endometrial thickness of the rat model. Therefore, infusion of BMSCs enhances endometrial cell regeneration probably through immunomodulatory and migratory processes.^60^ In addition, Wang et al^61^ have shown that BMSC transplantation is effectively repair the damage of endometrium, through upregulation of estrogen receptor (ER) and progesterone receptor (PR) expression.

In the clinical studies, Santamaria et al^62^ performed a transplantation of CD133^+^ BMSCs to the Asherman's syndrome and endometrial atrophy patients. BMSC infusion has the beneficial effects as demonstrated by increasing the density of mature vessels in the endometrium, improving the duration and intensity of menstruation. Strikingly, some patients established pregnancy without any medical intervention after treatment.

#### 1.5.2 Adipose‐derived stem cells

Adipose‐derived stem cells (ADSCs) can be isolated from adipose tissue. ADSCs have been intensively examined for the therapeutic efficiency due to the ease of accession of sample through liposuction. ASCs exhibit typical characteristics of MSCs including self‐renewal, differentiation ability, and immunomodulatory properties. For infertility treatment, several study demonstrated the promising results including endometrial infertility treatment.

In experimental rat model, the combination of ADSCs and estrogen treatment induced endometrial tissue regeneration.^63^ Shao et al^64^ convince that transplantation of ADSCs improves the endometrial injury repair through differentiation ability of ADSCs to endometrial cells. The authors transplanted green fluorescent protein (GFP)‐labeled ADSCs into the animal model and later detected GFP‐endometrial epithelial cells. ADSCs increased endometrial thickness, number of micro‐vessel and endometrial glands and subsequently improved the fertility of the animals. The authors additionally demonstrated the mechanism in which ADSCs improve endometrial condition via elevating the expression of ER alpha (ERα), ER beta (ERβ), and PR. Despite of using the ADSC itself, exosome secreted by ADSCs has been proved to be promising treatment for patient with IUAs and infertility. ADSC‐derived exosome can promote regeneration of endometrium, remodeling of collagen and expression of integrin‐β3, leukemia inhibitory factor (LIF), and vascular endothelial growth factor (VEGF).^65^

In clinical trial, Sudoma et al^66^ show the first cases of pregnancies and childbirths after autologous ASCs use for endometrium recovery. The endometrium thickness of 20 out of 25 patients was increased after ASCs sub‐endometrial transplantation. A total of 13 pregnancies occurred and 9 healthy babies were born.

#### 1.5.3 Umbilical cord‐derived stem cells

Human umbilical cord‐derived stem cells (hUC‐MSCs) can be isolated from cord tissue of the newborns. It is also known as Wharton's jelly MSCs. hUC‐MSCs express specific markers of MSCs including CD29, CD44, CD73, CD90, and CD105 but do not express CD34, CD45, and HLA‐DR.^62^ hUC‐MSCs exhibit high proliferation rate, low immunogenicity, differentiation potential, and prolong survival time after transplantation.^67^, ^68^, ^69^ Because of the safety and efficacy of using hUC‐MSCs, using clinical‐grade hUC‐MSCs to treat infertility patients is being studied and developed. In the animal model, Zhang and colleagues (2018)^70^ found that hUC‐MSCs can repair injured endometrium thereby increasing the number of implantation embryos, through downregulated proinflammatory and upregulated vascular factors. Other study demonstrated that intramuscular injection of hUC‐MSCs effectively treat uterine niches after cesarean delivery.^71^

In clinical trial, transplantation of hUC‐MSCs loaded onto a degradable collagen scaffold into the uterine cavity of IUA patients demonstrated the safety and efficacy of stem cell therapy and tissue engineering. After transplantation, hUC‐MSCs maintained their survival within the degenerated site of the endometrium. Not only these results indicate a promising future for IUA patients, but also prove that tissue engineering can be applied with hUC‐MSCs in order to increasing their therapeutic efficiency.^72^

#### 1.5.4 Amniotic tissue‐derived stem cells

Human amniotic tissue is a postnatal membrane, normally discarded after delivery. Human MSCs can be isolated from amniotic tissue called human amniotic‐derived stem cells (hAMSCs). Interestingly, human amniotic epithelial cells (hAECs) which are the epithelial cells derived from amniotic tissue exhibit multipotential differentiation, immune‐regulating potential, and low tumorigenicity similar to hAMSCs and other type of MSCs.^73^ Therefore, hAECs have a potential in cell‐based therapy.

In an IUA mouse models, Li et al^73^ showed that endometrial morphology was improved after transplantation of hAECs. hAECs increased endometrium thickness through stimulating of angiogenesis and proliferation of endometrial stromal cells. Moreover, hAECs activated autophagy pathway of the endometrium resulting in decreasing of fibrotic area of endometrium. Finally, infertility of the IUA mice was improved after stem cell transplantation. In an IUA rat models, the results revealed that mRNA levels of tumor necrosis factor‐α and interleukin‐1β which is proinflammatory cytokines were downregulated while basic fibroblast growth factor and interleukin‐6 which is anti‐inflammatory cytokines were upregulated after transplantation of hAMSCs. Therefore, transplantation of hAMSCs promotes endometrial regeneration, possibly due to immunomodulatory properties.^74^

#### 1.5.5 Menstruation‐derived stem cells

While MSCs isolated from bone marrow, amniotic fluid or adipose tissue is complicated due to the need of surgical procedure. It is necessary to find an alternative source of MSCs that easy to access. A recent study demonstrated that cells locates in basal layer of the endometrium exhibit stem cell characteristics including high proliferation, self‐renew, and differentiation potential.^75^ Direct access to endometrial stem cells by biopsy or cuvette may damage to the endometrium. Fortunately, the endometrial stem cells can be easily derived from menstruation. Menstruation‐derived MSCs (MenSCs) can be derived from the menstrual blood and exhibit MSC characteristics that meet the criteria of ISCT.^76^, ^77^

For application of MenSCs in endometrial abnormality, MenSCs survived in the endometrium of mouse model with endometrial damage after transplantation.^77^ Besides survival, MenSCs improved the embryo implantation via stimulating the expression of vimentin, keratin, and VEGF of the endometrium^77^ of increase the endometrial thickness via the regulation of protein kinase B signaling pathways.^78^ Protein kinase B plays a role in cell proliferation, metabolism, and survival.^79^ Interestingly, Zhang et al^80^ systemically compared the efficiency of three transplantation protocols including MenSCs, platelet‐rich plasma (PRP), combination of MenSCs and PRP and placebo control for treatment of intrauterine adhesion in a rat model. The results clearly demonstrated that MenSCs combined with PRP improved proliferation, angiogenesis, and morphological recovery of endometrium. The authors suggested that changing of Hippo signaling pathway, regulating Wnt5, Gdf5, downstream factors as well as connective tissue growth factor are the involved mechanism.^80^

In clinical trial, the promising results were obtained after transplantation of autologous MenSCs for severe Asherman's syndrome patients. Tan et al^81^ demonstrated that MenSCs increased the endometrial thickness and improved the pregnancy of severe Asherman's syndrome patients.^81^ These data open the new hope for infertility patients caused by Asherman's syndrome. Endometrial stem cells are ideal MSCs for the treatment of endometrial infertility. Moreover, MenSCs offer the ease of sample collection. Thus, application of MeMSC in management of endometrial infertility can be less invasive in comparison with other source of MSCs.

## 2 CONCLUSIONS AND FUTURE PERSPECTIVE

MSCs have exhibited a great potential for treatment of infertility caused by endometrial dysfunction. Adipose‐ and menstruation‐derived stem cells show advantages over other sources because the cells can be derived easily and do not causes graft rejection after autologous transplantation. Prior to be routinely applied the clinical setting, there are some bottleneck that has to be overcome. Firstly, most of the studied has been done using rodent as an animal model. The differences in uterine anatomy and physiology between human and rodent limited the results of animal models to be translated. However, a study in a larger animal like equine which shares anatomical and physiological similarity to human demonstrated a useful information.^82^, ^83^ The findings in equine could guide future clinical trials and treatment. Secondly, expansion of MSCs in most of the studies is mostly prepared in the small scale of academic laboratories resulting in enormous variability of protocols. To provide the clinical‐grade MSCs for therapeutic purposes, laboratory must be complained with GMP regulations. This will ensure the safety for the patients and avoid the complication of transplantation of MSCs. Lastly, in order to utilizing MSCs after transplantation, the combination of cell or tissue engineering such as scaffold, nanoencapsulation, or modification of MSCs has to be developed and applied with clinical‐grade MSCs. Taken altogether, MSCs will be efficiently and safely applied in clinical setting for treatment of endometrial infertility.

## CONFLICT OF INTEREST

There are no conflicts of interest to declare.

## HUMAN/ANIMALS RIGHTS

This article was performed without having any human or animal subjects.
